# Reviewed article: Perotto JJM, Fonseca MJM, Braga JU. Spatiotemporal analysis of tuberculosis incidence on the border between Brazil and Argentina: a time series study, 2009-2021. Epidemiol Serv Saude. 2025:34;e20240650

**DOI:** 10.1590/S2237-96222025v34e20240650.a

**Published:** 2025-10-20

**Authors:** Lucas Vinícius de Lima

**Affiliations:** 1 Universidade Estadual de Maringá, Maringá, PR, Brazil

## Peer review A

## Questionnaire

Does the manuscript contain new and significant information to justify publication? Yes

Does the Abstract (Summary) clearly and accurately describe the content of the article? Yes

Is the problem significant and concisely stated? Yes

Are the methods described comprehensively? No

Are the interpretations and conclusions justified by the results? Yes

Is adequate reference made to other work in the field? Yes

## Manuscript Structure

Length of article is: Adequate

Number of tables is: Adequate

Number of figures is: Adequate

Please state any conflict(s) of interest that you have in relation to the review of this paper. None

Interest: Good

Quality: Good

Originality: Avarage

Overall: Good

Recommendation: major Revision

## Comments

Prezados autores, o artigo aborda um tema relevante e atual ao investigar a incidência de tuberculose em uma região de fronteira internacional entre Brasil e Argentina, com foco em análises espaciais e temporais. No entanto, há pontos que precisam ser aprimorados para fortalecer a coesão, a clareza e o impacto do estudo. Destacam-se as seguintes recomendações:

Introdução:

- A transição entre parágrafos carece de fluidez. Uma melhor conexão lógica ajudaria a construir uma narrativa mais coesa, especialmente ao ligar a importância global da tuberculose com os os desafios locais de regiões fronteiriças.
- Evitar construções genéricas de frases que não trazem informações relevantes ou que podem ser sintetizadas. Por exemplo, a frase: “A tuberculose é uma doença infectocontagiosa que apesar de ser muito antiga permanece como um problema de saúde pública mundial a ser enfrentado” poderia ser substituída por uma versão mais objetiva, como: “A incidência de tuberculose, uma doença infectocontagiosa milenar, tem seu risco aumentado sobretudo por condições socioeconômicas deficitárias da população.”
- Atualizar os dados apresentados no segundo parágrafo para informações do Global Tuberculosis Report 2023 (disponível em: https://www.who.int/teams/global-tuberculosis-programme/tb-reports/global-tuberculosis-report-2024).
- Avaliar a construção de frases no terceiro parágrafo, tornando-as mais objetivas. Por exemplo, substituir “as áreas de fronteira geralmente se constituem em lugares importantes para transmissão da tuberculose” por uma frase mais técnica, como: “Devido às vulnerabilidades intrínsecas às áreas de fronteira - como mobilidade populacional, condições de vida inadequadas e acesso limitado aos serviços de saúde - essas regiões apresentam maior risco de transmissão da tuberculose”.
- Penso que os autores precisem utilizar uma linguagem mais técnica e objetiva. Por exemplo: a expressão “lugares importantes para transmissão” pode ser substituída por termos mais específicos, como “áreas de alta vulnerabilidade epidemiológica” ou “zonas de risco elevado para transmissão da tuberculose”.
- O trecho sobre a dificuldade no controle da tuberculose em áreas fronteiriças é pertinente, mas falta uma conexão mais clara com a literatura que discuta desafios estruturais e organizacionais dessas regiões, como subnotificação, mobilidade populacional, integração de sistemas de saúde, acesso e vinculação ao diagnóstico e tratamento, etc.
- Embora seja interessante contextualizar o cenário do estudo, acredito que essa informação mais específica, como a menção das cidades, possa ser apresentada na seção de métodos. Na introdução, sugiro manter o enfoque na região de fronteira e trazer estudos paralelos em outras cidades gêmeas que ressaltem a importância da avaliação e vigilância da tuberculose em diferentes cenários. Penso que o estudo não precise se justificar pela “inovação” em analisar os dados de um local específico, mas sim nas implicações para a saúde de indivíduos e coletivos, como um norte da saúde pública.
- A justificativa atual está centrada na especificidade geográfica do local, mas pode ser fortalecida ao destacar a relevância das análises temporais e espaciais para subsidiar políticas públicas transnacionais, alinhadas a metas globais como a End TB Strategy.

Métodos:

- Sugiro organizar a seção em subtópicos, permitindo maior clareza e compreensão das etapas seguidas. Por ser um estudo descritivo e ecológico, sugiro: Tipo de estudo, Contexto do estudo, População do estudo, Fonte de dados, Variáveis do estudo, Mensuração, Análise de dados, Questões éticas.
- Penso que o estudo não seja apenas ecológico. Embora o seu ponto forte seja a abordagem ecológica, com emprego de análises espaciais e de séries temporais, há um componente observacional descritivo, com a caracterização do perfil das pessoas acometidas. Sugiro que o desenho seja apresentado como “estudo descritivo e ecológico”, para maior alinhamento. Além disso, informações analíticas não devem ser antecipadas, como “com geocodificação das taxas médias de incidência”. Sugiro apenas especificar quais foram as unidades de análise do componente ecológico, sem se aprofundar nas medidas e análises.
- As bases de dados não foram mencionadas (SINAN no Brasil e a base da Argentina); faltam informações detalhadas sobre como esses dados foram acessados, sua completude e os critérios de seleção. Além disso, seria necessário mencionar se existiam dados ausentes e como eles foram trabalhados nas análises de perfil.
- A Figura 1 é interessante para apresentar o cenário do estudo, especialmente por abordar municípios de um outro país. Sugiro apenas mencioná-la junto aos parágrafos que contextualizam os locais, fazendo referência direta às partes A, B e C.
- Na definição de incidência, o Ministério da Saúde do Brasil considera não apenas os casos novos e as entradas como “não sabe”, mas também os casos de pós-óbito. Importante mencionar e justificar o porquê da não inclusão das pessoas com essa entrada.
- Sugiro detalhar qual foi o tipo de teste qui-quadrado empregado. Por inferência, considerando a natureza dos dados e o foco da análise, presume-se que tenha sido o de Pearson, mas existem outras estatísticas de qui-quadrado que poderiam ser utilizadas.
- Na definição da vizinhança, importante informar qual foi o critério utilizado (torre, rainha, etc.), bem como o nível de ordem considerado entre eles (primeira, segunda, etc.).
- As técnicas estatísticas e espaciais são listadas, mas a justificativa para sua escolha não está clara. Explicar por que foram utilizados o índice de Moran global e local, a regressão joinpoint e as taxas bayesianas empíricas. Por exemplo: “O índice de Moran local foi usado para identificar clusters espaciais, que são pertinentes para identificar áreas de risco para tuberculose”. Também seria interessante detalhar o que cada análise fornece de interpretação, destacando as diferenças entre elas, como forma de justificá-las.
- A descrição da regressão joinpoint não menciona como os períodos foram definidos ou se houve alguma consideração para tendências não lineares. Deve-se especificar como os pontos de junção foram determinados e interpretados (por exemplo: critérios para considerar mudanças estatisticamente significativas - método de permutações de Monte-Carlo, método quantil-empírico, entre outros).
- Acho que precisa ficar claro que o recorte temporal 2009-2019 e 2009-2021 foi utilizado apenas para a construção dos mapas de distribuição e autocorrelação espacial. E que para a análise de tendência temporal foi utilizado o período integral, tendo em vista que o método já proporciona essa segmentação, se houver significância estatística.

Resultados:

- A apresentação dos resultados é extensa e carece de uma organização mais clara entre os aspectos temporal, espacial e descritivo. Evitar repetir as informações já dispostas nas tabelas e figuras, preconizando apenas os dados mais relevantes.
- A explicação sobre as diferentes análises espaciais nos métodos se mostra ainda mais importante nos resultados, pois os mapas da Figura 2 parecem mostrar a mesma informação, de formas diferentes. Sugiro deixar mais claro o que cada técnica traz e como se difere das demais. Da forma atual, a informação está redundante.
- Na comparação direta entre as VPA do crescimento para Santa Catarina e Miosiones, é preciso deixar claro, nos métodos, se as variáveis dependentes foram logaritmizadas. Do contrário, a comparação entre VPA é inapropriada. Além disso, é importante mencionar como se deu a correção da autocorrelação serial das séries, visto que o software permite que o usuário faça esse controle. Se não tiver sido corrigido, deve-se pontuar como uma limitação.
- As figuras e tabelas poderiam ser mais exploradas para enriquecer a interpretação dos resultados. Por exemplo, incluir gráficos de tendências temporais que mostrem claramente a evolução da incidência por país e por períodos, em detrimento de tabela.

Discussão:

- A discussão precisa ser reformulada em sua totalidade. Os autores somente reapresentam os resultados e utilizaram poucos estudos, inclusive alguns repetidos da introdução, para corroborar os achados. É preciso avançar em reflexões e implicações práticas desses resultados para os serviços de saúde e para as políticas públicas na região fronteiriça estudada.
- A relevância do estudo não está suficientemente destacada. Talvez enfatizar como o estudo contribui para a compreensão da tuberculose em regiões fronteiriças, uma área frequentemente negligenciada na literatura.
- As comparações com estudos anteriores são superficiais e não exploram plenamente a convergência ou divergência dos resultados. Sugiro relacionar os achados sobre clusters espaciais e tendências temporais a estudos realizados em outras regiões de fronteira ou contextos urbanos similares. Ainda, explorar diferenças metodológicas entre este estudo e outros, discutindo como isso pode influenciar os resultados.
- A discussão dos clusters espaciais é limitada e carece de interpretações mais críticas. Uma proposta seria discutir possíveis razões para a concentração de clusters alto-alto na Argentina, como desigualdades sociais, infraestrutura de saúde e mobilidade populacional. Ainda, propor hipóteses sobre os padrões baixo-baixo no Brasil, considerando fatores como subnotificação, qualidade da vigilância ou diferenças demográficas.
- A análise do impacto da pandemia de covid-19 nos registros de tuberculose é mencionada, mas não explorada criticamente. Importante discutir como as políticas de saúde pública durante a pandemia podem ter afetado os registros nos dois países, levantando hipóteses para as tendências estacionárias.
- A discussão não explora suficientemente como os resultados podem subsidiar políticas de saúde pública. Seria interessante sugerir intervenções específicas baseadas nos clusters identificados, como campanhas de diagnóstico em áreas de maior risco ou esforços para melhorar a vigilância em áreas de subnotificação. Além disso, apontar a necessidade de cooperação transnacional para fortalecer o controle da tuberculose em áreas fronteiriças, incluindo ações bilaterais para monitoramento e tratamento.
- As diferenças nos sistemas de saúde dos dois países não são suficientemente analisadas. Deve-se discutir como as discrepâncias nos registros de tuberculose podem ser influenciadas por diferenças nos critérios de notificação, infraestrutura de saúde ou recursos disponíveis. Inclusive, explorar o impacto das condições socioeconômicas nos padrões observados, como a alta concentração populacional em áreas urbanas na Argentina.
- A conexão com metas globais, como a End TB Strategy, é vaga. Seria necessário discutir como os achados refletem desafios para atingir a meta de redução da incidência de tuberculose até 2035, sobretudo em cenários de fronteira entre países, dadas as diferenças estruturais entre eles.
- A discussão das limitações é breve e não aborda todos os possíveis vieses. É preciso discutir a dependência de dados secundários e a possibilidade de subnotificação, especialmente em áreas de baixa incidência. Ainda, abordar as diferenças metodológicas entre os sistemas de notificação dos dois países como um possível fator de viés (por exemplo, a própria definição de casos novos é diferente entre eles).
- As conclusões são limitadas a uma síntese dos achados, sem destacar suficientemente as implicações práticas. Sugiro focalizar os principais achados em relação à distribuição espacial e temporal, reforçando a necessidade de ações específicas para controle da tuberculose em áreas fronteiriças e a importância da cooperação internacional.

Como recomendações adicionais, penso que há uma desconexão entre introdução, métodos, resultados e discussão. A transição lógica entre o que se propõe investigar, como foi investigado e os achados precisa ser reforçada. Por exemplo, a introdução precisa definir claramente as questões de pesquisa e as hipóteses, conectando-as explicitamente às análises realizadas e aos resultados apresentados. Isto significa trazer maior peso para a importância das análises espaciais e temporais no contexto do estudo.

Como os resultados não abordam suficientemente outros fatores determinantes para a tuberculose, como desigualdade social, infraestrutura de saúde e migração populacional, a discussão precisa trazer elementos adicionais ou discutir dados complementares, como a correlação com indicadores socioeconômicos, de infraestrutura e de recursos humanos.

O enfoque transfronteiriço do estudo está diluído, sendo tratado mais como uma comparação entre Brasil e Argentina do que como uma análise integrada de uma região fronteiriça. É necessário reforçar a ideia de que a fronteira é um sistema único, explorando mais as implicações para colaboração binacional em vigilância e controle da tuberculose. Isso inclui discutir políticas existentes e lacunas em programas de saúde.

A conexão com os Objetivos de Desenvolvimento Sustentável (ODS) é superficial. Os autores precisam discutir como os resultados podem contribuir para o cumprimento do ODS 3 (saúde e bem-estar), especialmente no contexto de reduzir a carga de doenças infecciosas em populações vulneráveis. O estudo permite trabalhar com esse objetivo, mas ele não foi abordado em nenhum momento do texto.

Com esses ajustes, o estudo poderá contribuir tanto para a literatura quanto para intervenções no controle da tuberculose. Dessa forma, penso que o manuscrito apresenta potencial de publicação na Revista Epidemiologia e Serviços de Saúde, sobretudo pela originalidade, relevância e profundidade. No entanto, um aprofundamento maior em todas as seções do estudo, inclusive uma descrição mais robusta dos métodos, é necessária para a qualificação do material. 

